# Nutrition Education “Shorts”: The Effect of Short-Form Media on Conveying Information About Improving Diet Quality

**DOI:** 10.3390/nu17101612

**Published:** 2025-05-08

**Authors:** Gail C. D’Souza-Rushton, John W. Long, Amber Denmon, Penny M. Kris-Etherton, Kristina S. Petersen, Travis D. Masterson

**Affiliations:** 1 Department of Nutritional Sciences, Pennsylvania State University, University Park, PA 16802, USA; jwl6179@psu.edu (J.W.L.); pketherton@gmail.com (P.M.K.-E.); kup63@psu.edu (K.S.P.); travis.d.masterson@psu.edu (T.D.M.); 2 Penn State Extension, Pennsylvania State University, University Park, PA 16802, USA; azd241@psu.edu

**Keywords:** nutrition education, herbs and spices, short videos, diet quality, cooking

## Abstract

Background: The 2020–2025 Dietary Guidelines for Americans recommends using herbs and spices in cooking to decrease salt, added sugars, and saturated fats (SFA). To promote a healthier eating pattern, there is a need to teach consumers how to do this. Objective: To compare the effectiveness of five short (~1 min) nutrition education videos to five longer (~5 min) videos by evaluating participants’ interest, knowledge, confidence, and likeliness to use herbs and spices in their cooking before and after the videos. To evaluate participant perceptions of the videos and barriers to using herbs and spices in cooking. Methods: We conducted a cross-sectional study using a sample of American adults through Dynata. Pre-and post-survey questions inquired about participants’ interest, knowledge, confidence, and likeliness to use herbs and spices in cooking. Participants were randomized to view either short or longer videos that included recipe instructions on how to use herbs and spices in cooking. The content of both videos was the same but the format (short and longer video included ingredients and directions while the longer video also included food safety and handling) and duration differed. The videos featured recipes that were lower in salt, added sugars, and SFAs, and herbs/spices were used as a replacement. We also evaluated participants’ perceptions of the videos. Results: Participants (*n* = 201) were 59% female, 80% White, and had a mean age of 51 (SD = 15) years. All participants reported higher interest, knowledge, confidence, and likeliness to use herbs and spices in cooking after watching the short and longer videos (*p* < 0.05). Furthermore, participants reported that both short and longer videos were interesting, engaging, conveyed educational information, were easy to follow and understand, and an effective method to increase the use of herbs and spices in cooking. Our analysis revealed no statistically significant differences between short vs. longer videos. Conclusions: Overall, both short and longer videos appeared to have a similar impact on consumer interest, knowledge, confidence, and likeliness to use herbs and spices in cooking. Future research should assess changes in dietary intake in response to the videos.

## 1. Introduction

Over the last few decades, eating habits in the United States have led to overconsumption of salt, added sugars, and saturated fats (SFAs) [1]. The relationship between increased consumption of salt, added sugars, and SFAs and adverse health outcomes has been studied for many years with unhealthy eating habits being associated with multiple chronic diseases [2]. Cancer, cardiovascular disease, type 2 diabetes, and obesity have all been linked to poor diet quality, which can lead to other comorbidities and multimorbidity interactions [3].

Previous studies have recommended at-home cooking as a method to improve adherence to healthy eating, which is associated with lower risks of incident cardiovascular disease, cardiovascular mortality, and all-cause mortality among US adults [4,5]. At-home cooking helps individuals control ingredients in foods, leading to overall better diet quality [6,7]. Unfortunately, some barriers to at-home cooking in American households include affordability and lack of time, which further impact diet quality [8]. To improve diet quality, the 2020–2025 Dietary Guidelines for Americans (DGA) recommends using herbs and spices to decrease salt, added sugars, and SFAs in cooking [9]. By incorporating herbs and spices in at-home cooking, individuals can enhance the flavors of foods according to their preference, without compromising taste in meals [10,11]. Additionally, incorporating a high dosage of herbs and spices to an American diet has shown improvements in blood pressure in adults with an elevated risk of cardiometabolic disease [12]. A recent study by Lawler et al. surveyed adults who viewed nutrition education videos that recommended herb and spice usage as a means to reduce their salt, added sugars, and SFA intake in at-home cooking. Participants from this study reported an increase in knowledge, confidence, and likeliness of using herbs and spices in their cooking after viewing the videos [11]. The videos shown in this study were effective in recommending herb and spice usage, but the videos were 3–5 min in length, which is longer than the short-form video duration we intended to use in our study, such as TikTok, Instagram Reels, and YouTube Shorts.

Previous studies have also highlighted the importance of format and duration of educational videos and the impact on individual engagement and response [13,14]. For example, a study by Adam et al. assessed the impact of 4–6 min cooking instruction videos on improving eating behaviors among people in different countries [15]. Participants from this study reported positive changes in eating behaviors over time and significant increases in their perceived health after viewing the online cooking videos [15]. Similarly, a study comparing the effectiveness of video vs. handout educational materials among family medicine clinic patients found that videos were superior to handout materials when educating patients about herb use in meal preparation. Providing video-based education can be a useful tool in waiting rooms, minimizing the burden placed on physicians to educate patients on healthy eating [16].

According to the 2024 HubSpot State of Marketing Report, short-form video content has been effective in retaining individual attention, with videos under 60 seconds (on social media platforms like TikTok, Instagram Reels, and YouTube Shorts) being preferred among current consumers [17]. With the rise in decreasing attention spans of consumers, and copious media consumption, especially among the younger generations, short-form video content is found to enhance engagement and is better liked when compared to traditional long-format videos [17,18]. The preference for short-form video content can be used as a means to overcome current public health communication challenges by adapting to new and evolving methods of public health communication [19]. Additionally, short-form video content is considered better than long-form content due to the short delivery time, lower production costs, and the opportunity of engaging with the audience in a creative way especially in our current fast-paced environment [19,20].

Therefore, the purpose of this study was to compare the effectiveness of five short-form (~1 min/video) nutrition education videos to five longer (~5 min/video) previously research-tested nutrition videos [11]. Specifically, consumers were asked to evaluate their interest, knowledge, confidence, and likeliness to use herbs and spices in their cooking before and after watching the nutrition education videos [11]. Participants were also asked to report their perceptions of the videos. Finally, this study also assessed if there were perceived potential barriers to adopting the use of herbs and spices in their cooking after viewing the content. We hypothesized that the short nutrition education videos would be more effective in increasing participants’ interest, knowledge, confidence, and likeliness to use herbs and spices in their future cooking. We further hypothesized that the short videos would be rated higher in interest, knowledge, and confidence in using herbs and spices in cooking when compared to the longer nutrition education videos.

## 2. Materials and Methods

Study participants were recruited using a third-party market research agency, Dynata (Shelton, CT, USA). Email invitations with a survey link were sent to a pool of potential participants through panels and intercepts. Panels consisted of databases of potential participants who declared cooperation with future data collection in exchange for a reward/incentive. Data collection through intercepts occurred when potential participants were asked to take a survey for a reward while they played a game or read the news. Upon accessing the survey link, participants were then screened for study inclusion and exclusion criteria. Inclusion criteria were being at least 18 years of age, could read and write in English, and reportedly had cooked a meal at home on at least 2 days in the last week. Eligible participants received the summary of the research and provided their implied consent by checking a box that indicated they read and understood the information presented, and also, they voluntarily wished to continue with the survey. All eligible participants then completed a pre-intervention survey.

Participants were blind randomized an odd or even number to their REDCap record when they signed up for the study through Dynata, a market research agency. If participants were assigned an odd number, they viewed the five short nutrition education videos and if they were assigned an even number, they viewed the five long nutrition education videos (see video materials for details). After viewing the videos, participants completed a post-intervention survey. All data were collected via the Research Electronic Data Capture (REDCap) program. REDCap is a secure, web-based application designed to support data capture for research studies [21]. The Pennsylvania State University Institutional Review Board approved the study protocol (Protocol #- STUDY23901). Participants were compensated for their time according to standard rates used by Dynata.

### 2.1. Measures

#### 2.1.1. Pre-Intervention Survey

Participants completed a demographic questionnaire and self-reported their age, height, weight, sex, gender, race, ethnicity, income, and education level. Participants were also asked a series of questions in which they rated their interest, knowledge, confidence, and likeliness to use herbs and spices while preparing a meal before viewing the educational videos (see Supplementary Table S1). These questions have been utilized in previous studies inquiring about the use of herbs and spices in daily cooking [11]. All interest, knowledge, and confidence response options were represented on a 5-point Likert scale of 0 (extremely disinterested) to 5 (extremely interested). Participants then indicated their likeliness to use herbs and spices to enhance the taste and consumption of vegetables using a 5-point Likert scale of 0 (very unlikely) to 5 (very likely). Participants were also asked if they were in favor of using herbs and spices in their food, and if herbs and spices fit easily into their cooking with response options represented on a 5- point Likert scale of 0 (strongly disagree) to 5 (strongly agree). All questions about participants’ usage of herbs and spices in their cooking were developed using Proctor’s implementation framework outcome measures for evaluation purposes [22]. Proctor’s outcome measures serve as indicators of outcome success and offer a theoretical framework basis for our study [22]. The outcome measures used in our study were acceptability, adoption, and appropriateness. All questions appear in Supplementary Table S1.

#### 2.1.2. Video Materials

The original video materials were developed for a previous study with complete development methods reported elsewhere [11]. Nutritional scientists, Penn State Extension educators, and professional chefs created recipes using herbs and spices to reduce the use of salt, added sugars and SFAs when preparing food at home. The recipes were converted into videos with WPSU featuring a Penn State Extension educator who is also a Registered Dietitian Nutritionist (RDN) [23]. The first video taught participants how to create a no-salt spice blend, while the subsequent four videos showed recipes using the blend and other spices. After the creation of the longer (~5 min) videos, Penn State Extension educators, the study team, and WPSU worked together to adapt the content to fit short-form content on social media. The researchers reviewed the content in the longer videos that were critical to using herbs and spices in the recipes in place of salt, added sugars, and SFAs and created the short videos to convey the same message. According to YouTube Shorts, short-form content videos can be up to 60 seconds in length [24]. The final five short videos totaled 4 min and 33 s (~55 s) while the five longer videos totaled 17 min and 5 s (~3 min and 25 s). All videos are publicly accessible at https://www.myplate.gov/partner-resources (accessed on 5 February 2024).

#### 2.1.3. Post-Intervention Survey

After viewing the educational videos, the post-intervention survey asked participants to rate their interest, knowledge, confidence, and likeliness to use herbs and spices while preparing a meal (similar to the pre-intervention survey) (see Supplementary Table S1) [11]. The post-intervention survey asked participants about potential barriers to using herbs and spices when cooking with response options provided based on a literature review [25,26,27,28]. Participants were then asked to indicate if each item was “not a reason”, a “minor reason”, or a “major reason”. Some of the eight options were, “I feel that herbs and spices have distinctive flavors that are too strong for me”, “I think herbs and spices are expensive to purchase”, and “I do not have access to herbs and spices where I live” (see Supplementary Table S1). Participants also evaluated the quality of the videos using a 5-point Likert scale of 0 (strongly disagree) to 5 (strongly agree). Some of the response options were, “the nutrition education videos conveyed educational information about the use of herbs and spices when cooking”, “the nutrition education videos were easy to follow and understand”, and “the nutrition education videos were interesting and engaging”. All questions appear in Supplementary Table S1.

#### 2.1.4. Statistical Analysis

Participants who did not complete all surveys and did not meet the inclusion criteria were excluded from the analysis. Means and frequencies were calculated to present sample characteristics from the demographic survey. Linear mixed effect models were used to assess random effects and fixed effects of time, randomization, age, education, sex, income, and the interaction between time and randomization [29]. Outcomes measured were interest, knowledge, confidence, likeliness variables, time (pre vs. post), video condition (short vs. longer), and the interaction between time and video condition. Means of all variables for interest, knowledge, confidence, and likeliness were used to calculate overall interest, knowledge, confidence, and likeliness of herb and spice usage [11]. To assess potential barriers to why some participants may not have wanted to or may have been unable to use herbs and spices in their cooking, frequencies and percentages for each category were calculated. Mean values were calculated to perform *t*-tests comparing participant evaluations of the nutrition education videos based on their length [16]. For all comparative tests, a *p* < 0.05 was considered statistically significant. All statistical analyses were conducted using R version 4.3.2 [30].

## 3. Results

A total of 290 participants responded to our initial survey request. Among them, 201 participants completed the survey and were included in the analysis. The demographic characteristics of participants are summarized in Table 1.

### 3.1. Changes in Interest, Knowledge, Confidence, and Likeliness in Using Herbs and Spices

The results of the linear mixed models investigating differences in interest, knowledge, confidence, and likeliness to use herbs and spices are summarized in Table 2. Our analysis revealed a statistically significant main effect of time (pre-intervention vs. post-intervention) for overall interest, overall knowledge, overall confidence, and overall likeliness (*p* < 0.05 for all). There was no statistically significant interaction between time and condition (short vs. long videos) for any of these variables (all *p* > 0.05) other than overall confidence (*p* = 0.01).

The models indicated interactions for three of the individual variables: (a) confidence in using herbs and spices to decreased use of added sugar (β = 0.38, *p* = 0.01265), (b) confidence in using herbs and spices to decreased use of SFAs (β = 0.32, *p* = 0.03081), and (c) likeliness to use herbs and spices to improve the taste and consumption of vegetables (β = 0.24, *p* = 0.0364). In models where there were no interactions, all variables had a main effect of time (pre vs. post) (*p* < 0.05 for all), except for the variable, “interest in the flavor profile of herbs and spices” (*p* = 0.073). Additionally, only one variable, “knowledge in making food healthier by flavoring foods/meals with herbs and spices” had a main effect on condition (short vs. longer) (β = −0.48, *p* = 0.03580). This analysis revealed that participants who viewed the longer videos experienced a significant reduction in their knowledge regarding making food healthier by flavoring foods/meals with herbs and spices. The results are presented in Table 2 and visualized in Supplementary Figures S1 and S2.

### 3.2. Participant Perceptions of Nutrition Education Videos

Participants rated the videos on a Likert scale from 1 (strongly disagree) to 5 (strongly agree) with average scores exceeding 4 (agree) across all evaluation metrics. Those who viewed the longer videos rated them higher and reported that the videos provided them with relevant information on how to increase the use of herbs and spices in their cooking (4.31 vs. 4.47, *p* = 0.02). No other statistically significant differences were observed (See Table 3).

### 3.3. Incorporating Herbs and Spices in Cooking

**Figure 1:**
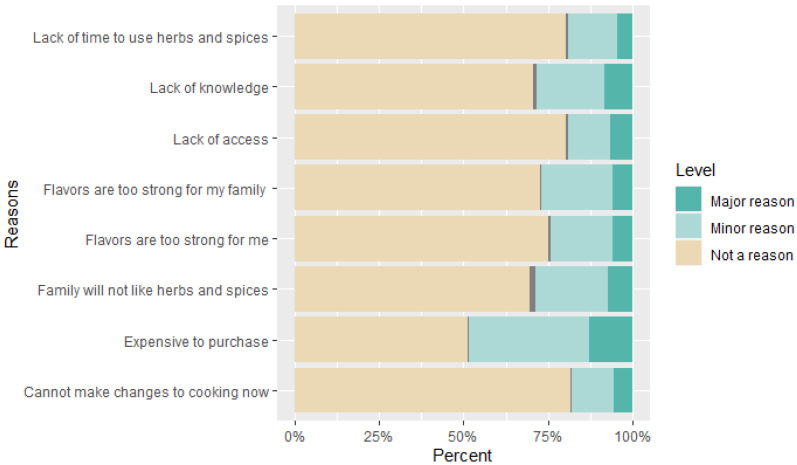
Reasons why people may not want to or may be unable to use herbs and spices in their cooking (*n* = 201).

Participants also reported reasons why they may not want to or may be unable to use herbs and spices in their cooking. Major reasons indicated by participants were because “herbs and spices are expensive to purchase” (13%), followed by “I do not have the knowledge on how to use herbs and spices in my cooking” (9%), and finally, “I feel that the people I cook for will not like herbs and spices being added to foods and meals (8%)” The complete list of results is presented in Figure 1.

## 4. Discussion

Previous studies have highlighted the importance of improving diet quality, particularly through an increase in consumption of healthy fats and vegetables to reduce cardiovascular disease and other conditions like depression [4,31,32,33]. One method of improving diet quality is by cooking at home to control for ingredients according to an individual’s dietary and nutritional needs [7,34]. Furthermore, incorporating herbs and spices into home-cooked meals can help enhance flavors while also helping in improving health by potentially reducing salt, added sugars, and SFAs [11,35].

The results of this study demonstrated that participants’ interest, knowledge, confidence, and likeliness to use herbs and spices increased after watching the nutrition education videos, irrespective of length. We hypothesized that the short videos would be more effective in increasing participants’ interest, knowledge, confidence, and likeliness to use herbs and spices in their future cooking, but contrary to our expectations, both short and longer videos were effective. Potential reasons for why the short videos did not outperform the longer videos could be the equally effective nature of both video formats since the content was identical, or participants’ cooking experience and their familiarity with using herbs and spices in their cooking. Participants evaluated both the short and longer videos and had similar positive feelings about them, with no statistically significant differences between the evaluations, except for confidence in using herbs and spices. Participants who viewed the longer videos experienced a significant reduction in their knowledge regarding making food healthier by flavoring foods/meals with herbs and spices. This finding could be due to a large amount of knowledge provided via the videos that may have overwhelmed participants who prefer the short-form video content. Finally, participants identified the cost of spices, knowledge on using herbs and spices, and their families’ preferences for herbs and spices as barriers in utilizing spices to replace salt, added sugars, and SFAs in their cooking.

After watching the videos, participants reported an increase in interest, knowledge, confidence, and likeliness of using herbs and spices in their cooking. Our study suggests that both short and longer videos are effective methods of educating people about the use of herbs and spices; although short videos are reportedly easier to create, cost-effective, and more engaging [36]. Short videos can provide the same information in a smaller amount of time and maintain participant engagement [37]. As seen with the rise in current media such as TikTok videos, Instagram stories, and YouTube shorts, the general public seems to enjoy short videos as they meet the demands of knowledge sharing in today’s fast-paced way of life [37,38]. Since short videos often convey the same message and are a more popular and preferred communication strategy in today’s world, it may be beneficial to create short nutrition education videos moving forward. A previous study assessing the effect of video technology on cooking self-efficacy among college students also reported an increase in confidence in cooking skill if the recipes were short and simple [39]. Similarly, in a medical clinic setting, patients typically spend a maximum of 8 min in the waiting room, which presents an ideal opportunity to share educational materials with them before their appointments begin [16]. Thus, short nutrition education videos can be used as a means to educate the general public about herb and spice usage without compromising content, whilst consumers can save, share, and review videos as needed.

Participants who watched the short or longer videos found the videos to be interesting, engaging, and helpful in learning about herbs and spices. While we initially hypothesized that the short videos would be more effective, our analysis did not reveal a statistically significant difference between the two formats. This lack of difference may stem from some participants, particularly those less experienced in cooking, finding the longer videos more beneficial as they provided more in-depth information on using herbs and spices in recipes. Thus, the effectiveness of the video materials may be related to one’s ability to cook, regardless of video length [40]. Additionally, the longer videos were only 5 min or shorter in length, which is relatively short when compared to a 10 or 20 min nutrition education video. Also, since we did not present both video formats to each participant, future studies should explore preferences for video format within the same population to gain a clearer understanding of effectiveness.

Our study also highlighted potential barriers to why participants may not want to or may be unable to buy herbs and spices to use in their cooking. The majority of participants highlighted that a major reason for them to not use herbs and spices was because herbs and spices were expensive, participants had no knowledge on how to use herbs and spices, and the people they cook for will not like herbs and spices. With rising food costs globally, the cost of purchasing herbs and spices, but not using them as often when cooking at home, may not justify purchasing them [41]. Additionally, using nutrition education videos similar to our videos to educate participants about herbs and spices has already demonstrated increased interest, knowledge, confidence, and likeliness in incorporating herbs and spices in everyday cooking [11]. Finally, everyone may not enjoy the taste of herbs and spices but educating people on how to cook at home using spices in their daily meals can help consumers test new spices to find something they enjoy.

There were limitations present in this study. For example, this was a cross-sectional study and did not follow participants over time to evaluate their future herb and spice usage. Future studies would benefit from conducting a follow-up or a longitudinal study to assess the herb and spice usage and diet quality among the study population over time to draw more robust conclusions from the impact of video education on their everyday cooking. Since both video formats were not shown to every participant to determine which was preferred, future studies should explore video format preferences in the same group to better understand their effectiveness. This study was conducted in a sample that reports at-home cooking, and the majority of the sample were of White racial background (~80%) so there may be limitations in generalizability. Future research should survey individuals that do not cook at home and target more diverse populations inclusive of other races and ethnicities. Participants from our study responded about the barriers to using herbs and spices in everyday cooking, but more information is needed to determine the reasoning for their opinions. Future research should include qualitative open-ended interviews with participants that capture information regarding their barriers and facilitators for spice usage. Our study mostly focused on the effectiveness of the length of videos on herb and spice usage, but future research should assess the quality of videos and content favored by participants such as content from other cuisines, health or taste focus of the video messaging, and factors that engage the consumer with the video. There were many strengths from this study. Although this was not a cross-over design study, this study helped prevent dropouts from only watching the short or longer videos and reduced the bias of watching the short video vs. the longer video first. This study is among the first to compare short and longer length nutrition education videos in a representative US sample. Additionally, data for this study was obtained through Dynata, and was not derived from a known sample or population. Finally, both the videos (short vs. longer) were highly ranked by all participants, and participants were more likely to use herbs and spices in the future compared to before watching the nutrition education videos.

## 5. Conclusions

Our research supports the use of nutrition education videos to educate the general public on using herbs and spices in daily cooking. Moving forward, the focus should be on creating short, more impactful videos that provide people with a clear message on how to use herbs and spices in their daily cooking. With the rise in short videos that facilitate engagement on social media such as Tiktok videos, Instagram stories, and YouTube shorts, public health practitioners and digital content creators can use short-form video formats to deliver engaging educational information to the general population. Short-form video formats can enable easy sharing of videos and rapid dissemination of nutrition education in a shorter amount of time when compared to longer nutrition education videos. Such videos may serve as a viable method of public health message dissemination and may also be valuable to drive engagement between patients and clinicians [16]. Future research should use nutrition education videos to educate people on herbs and spices using distinct types of messaging strategies to find the most effective messaging strategy for better learning outcomes.

## Figures and Tables

**Table 1:** Sample characteristics of participants who viewed short and long nutrition education videos.

Characteristic	Overall (*n* = 201)	Short Videos (*n* = 106)	Long Videos (*n* = 95)
Mean Age (SD)	51 (15)	50 (15)	51 (16)
Self-reported BMI, kg/m^2^ (SD)	27 (8)	27 (7)	28 (9)
Biological Sex			
Male	83 (41.29%)	42 (39.62%)	41 (43.16%)
Female	118 (58.71%)	64 (60.38%)	54 (56.84%)
Gender			
Male	79 (39.30%)	39 (36.79%)	40 (42.11%)
Female	119 (59.20%)	65 (61.32%)	54 (56.84%)
Transgender Male	1 (0.50)	1 (0.94%)	0
Transgender Female	0	0	0
Gender Variant/Non-Conforming	0	1 (0.94%)	1 (1.05%)
Not listed	0	0	0
Ethnicity			
Hispanic or Latino	12 (5.97%)	4 (3.77%)	8 (8.42%)
Not Hispanic or Latino	189 (94.03%)	102 (96.23%)	87 (91.58%)
Race			
American Indian	1 (0.50)	1 (0.94%)	0
Alaska Native	0	0	0
Asian	10 (4.98%)	6 (5.66%)	4 (4.21%)
Black or African American	30 (14.93%)	14 (13.21%)	16 (16.84%)
Native Hawaiian or Other Pacific Islander	0	0	0
White	160 (79.6%)	85 (80.19%)	75 (78.95%)
Current Pre-tax Household Income			
$0–$9999	15 (7.46%)	1 (0.94%)	14 (14.74%)
$10,000–$19,000	15 (7.46%)	10 (9.43%)	5 (5.26%)
$20,000–$49,999	63 (31.34%)	42 (39.62%)	21 (22.11%)
$50,000–$99,999	67 (33.33%)	34 (32.08%)	33 (34.74%)
$100,000 or more	41 (20.40%)	19 (17.92%)	22 (23.16%)
Education Level			
Some High School	2 (1.00%)	1 (0.94%)	1 (1.05%)
High School Diploma or GED	59 (29.35%)	30 (28.30%)	29 (30.53%)
Trade School	14 (6.97%)	9 (8.49%)	5 (5.26%)
Associate degree	39 (19.4%)	19 (17.92%)	20 (21.05%)
Bachelor’s degree	58 (28.86%)	32 (30.19%)	26 (27.37%)
Master’s degree	24 (11.94%)	11 (10.38%)	13 (13.68%)
Ph.D. or Professional Degree	5 (2.49%)	4 (3.77%)	1 (1.05%)

**Table 2:** Linear mixed models for the interest, knowledge, confidence, and likeliness of herb and spice usage pre- and post-viewing the short vs. longer nutrition education videos (*n* = 201).

		Time (Pre vs. Post)		Condition (Short vs. Longer)		Interaction	
		β	*p*-Value	β	*p*-Value	β	*p*-Value
Overall interest		0.27	**<0.0001**	0.00	0.97	−0.05	0.41
Interest in	Flavor profile of herbs and spices	0.12	0.073	0.00	0.99	−0.03	0.78
	Incorporating herbs and spices into cooking	0.14	**0.018**	0.43	0.78	−0.03	0.77
	Using herbs and spices to increase consumption of healthier foods	0.24	**0.003**	−0.07	0.71	0.03	0.81
	Using herbs and spices to decrease use of salt in cooking	0.38	**<0.0001**	−0.02	0.92	0.00	0.99
	Decrease use of added sugar in cooking	0.40	**<0.0001**	0.32	0.15	−0.20	0.12
	Decrease use of saturated fat in cooking	0.36	**<0.001**	0.15	0.51	−0.14	0.31
Overall knowledge		0.22	**0.001**	−0.15	0.11	0.17	0.08
Knowledge in	Using herbs and spices preparing foods or a meal	0.14	**0.040**	−0.16	0.35	0.08	0.43
	Making food healthier by flavoring foods/meals with herbs and spices	0.30	**0.002**	−0.48	**0.036**	0.26	0.065
Overall confidence		0.24	**0.001**	−0.25	**0.01**	0.27	**0.01**
Confidence in using herbs and spices to:	Decrease use of salt	0.26	**0.004**	−0.41	0.060	0.23	0.083
	Decrease use of added sugar	0.17	0.10	−0.69	**0.006**	0.38	**0.013**
	Decrease use of saturated fat	0.30	**0.003**	−0.64	**0.011**	0.32	**0.031**
	Increase palatability and consumption of healthier foods	0.21	**0.020**	−0.35	0.10	0.14	0.28
Overall likeliness		0.26	**<0.001**	−0.12	0.20	0.05	0.64
Likeliness to use herbs and spices	As a substitute for salt	0.25	**0.007**	−0.02	0.94	−0.14	0.28
	As a substitute for added sugar	0.42	**<0.001**	−0.15	0.56	0.04	0.79
	As a substitute for saturated fat	0.42	**<0.0001**	−0.16	0.52	0.03	0.83
	To improve taste and consumption of vegetables	−0.06	0.48	−0.37	0.060	0.24	**0.036**

The bold used are meant to indicate significant *p* values for time, condition and interaction.

**Table 3:** Participant evaluation of short vs. long nutrition education videos (*n* = 201).

Question	Short Video Group (*n* = 106) Mean	Long Video Group (*n* = 95) Mean	*p*-Value
The nutrition education videos			
Conveyed educational information about the use of herbs and spices when cooking ^1^	4.47	4.47	0.97
Provided video content in a concise manner regarding the use of herbs and spices when cooking ^1^	4.35	4.46	0.14
Provided me with credible information on how to use herbs and spices when cooking ^1^	4.48	4.53	0.49
Were easy to follow and understand ^1^	4.52	4.54	0.78
Provided me with relevant information on how to increase the use of herbs and spices in my cooking ^1^	4.31	4.47	**0.02**
Encouraged me to use herbs and spices in my cooking moving forward ^1^	4.48	4.42	0.43
Were an effective method to help increase the use of herbs and spices in my cooking ^1^	4.43	4.45	0.73
Were interesting and engaging ^1^	4.38	4.35	0.76

^1^ Likert scale values were 1 (strongly disagree) to 5 (strongly agree). The bold indicates significant *p* value for time, condition and interaction.

## Data Availability

Data described in the manuscript, code book, and analytic code will be made publicly and freely available without restriction at Open Science Framework: https://osf.io/cw9zd/?view_only=224be2f0320843399bf3f61afdeb6f8c (accessed on 4 December 2024).

## Supplementary Materials

The following supporting information can be downloaded at: https://www.mdpi.com/article/10.3390/nu17101612/s1, Table S1: Study questionnaire; Figure S1: Mean scores of interest, knowledge, confidence, and likeliness in participants who viewed the short vs. longer nutrition education videos (*n* = 201). There was a statistically significant main effect of time (pre-intervention vs. post-intervention) for overall interest, overall knowledge, overall confidence, and overall likeliness (*p* < 0.05 for all); indicated as *. There was no statistically significant interaction between time and condition (short vs. long videos) for any of these variables (all *p* > 0.05) other than overall confidence (*p* = 0.01); indicated as **. Results indicate that the effects of the intervention were not dependent on the length of the videos for any of the variables besides overall confidence; Figure S2: Participants’ likeliness to use herbs and spices as a substitute for salt, added sugars, saturated fat, and to improve taste and consumption of vegetables when cooking (*n* = 201). ^a^ The linear mixed model indicated a main effect of time (pre vs. post) (*p* < 0.05) for likeliness to use herbs and spices as a substitute for salt, added sugars, and saturated fat. ^b^ The linear mixed model also indicated an interaction between time (pre vs. post) and condition (short vs. longer videos) for likeliness to use herbs and spices to improve the taste and consumption of vegetables (β = 0.24, *p* = 0.0364).
