# Supplementary material for: Nutrition Education “Shorts”: The Effect of Short-Form Media on Conveying Information About Improving Diet Quality
Source: Nutrients. 2025 May 8;17(10):1612. doi: 10.3390/nu17101612 (PMC12114486; doi:10.3390/nu17101612)
Supplement: Supplementary file 1 [file nutrients-17-01612-s001.zip › Supplementary Table_S1.pdf]

	#	Variable / Field Name	Field Label<br>*Field Note*	Field Attributes (Field Type, Validation, Choices, Calculations, etc.)
Instrument: **Screening Questions** (screening_questions) Enabled as survey				
	1	[record_id]	Record ID<br>*Your User ID is the ID that was sent to you via e-mail.*	text
	2	[psid]	Dynata PSID	text, Required<br>Field Annotation: @HIDDEN 1111 //[userrole-id]
	3	[screen_age]	Are you over the age of 18?	yesno, Required<br>1 Yes<br>0 No
	4	[ineligible]<br><br>Show the eld ONLY if: [screen_age] = '0'	We are sorry, you are not eligible to participate in this study. Thank you for your time.	descriptive
	5	[screen_language]<br><br>Show the eld ONLY if: [screen_age] = '1'	Are you uent in the English language?	yesno, Required<br>1 Yes<br>0 No
	6	[ineligible_2]<br><br>Show the eld ONLY if: [screen_language] = '0'	We are sorry, you are not eligible to participate in this study. Thank you for your time.	descriptive
	7	[screen_cook]<br><br>Show the eld ONLY if:<br>[screen_age] = '1' and [sc reen_language] = '1'	In the last week, how many days (0-7) did you cook a meal at home?	dropdown<br>0 0<br>1 1<br>2 2<br>3 3<br>4 4<br>5 5<br>6 6<br>7 7
	8	[ineligible_3]<br><br>Show the eld ONLY if:<br>[screen_cook] = '0' or [scr een_cook] = '1'	We are sorry, you are not eligible to participate in this study. Thank you for your time.	descriptive
	9		Section Header: *Form Status*	dropdown

		[screening_questions_complete]	Complete?	0 Incomplete<br>1 Unveri ed<br>2 Complete
Instrument: **Consent Form** (consent_form) ? Enabled as survey				
	10	[consent_form]<br><br>Show the eld ONLY if:<br>[screen_age] = '1' or [screen_language] = '1' or [screen_cook] = '3' or [screen_cook] = '4' or [screen_co ok] = '5' or [screen_cook] = '6' or [screen_cook] = '7' or [screen_cook] = '2'	Section Header: *Please review the attached document. This link will take you to a new browser tab. After reviewing the document, you will have to return to this page to complete the survey.*	descriptive<br>(Attachment: SIM_Consent Form_12.1.pdf,<br>Display format: Link)
	11	[consent_box]<br><br>Show the eld ONLY if:<br>[screen_age] = '1' or [screen_language] = '1' or [screen_cook] = '3' or [screen_cook] = '4' or [screen_co ok] = '5' or [screen_cook] = '6' or [screen_cook] = '7' or [screen_cook] = '2'	{consent_form}<br>*This form corresponds with your written consent to participate in this research study.*	checkbox, Required<br>1 consent_box___1 By checking this box, you indicate that you have read and understand the information presented here, and you voluntarily wish to continue.<br>Custom alignment: RH<br>Field Annotation: @INLINE
	12	[consent_form_complete]	Section Header: *Form Status*<br><br>Complete?	dropdown<br>0 Incomplete<br>1 Unveri ed<br>2 Complete
Instrument: **Demographics** (demographics) ? Enabled as survey				
	13	[age]	What is your age? (in years)	text (number, Min: 18), Required
	14	[ineligible_age]<br><br>Show the eld ONLY if:<br>[age]<18	We are sorry, you are currently ineligible to participate in this study. Thank you for your time.	descriptive
	15	[height_selfreport_ft]	What is your current height? Feet {demo_feet} Inches {demo_inches}	descriptive
	16	[demo_feet]	Feet	text (number, Min: 4, Max: 7)
	17	[demo_inches]	Inches<br>*inches*	text (number, Min: 0, Max: 11), Required

	18	[demo_height]		calc<br>Calculation: ([demo_feet]*12)+[demo_inches]<br>Field Annotation: @HIDDEN-SURVEY
	19	[weight_selfreport]	What is your current weight ?<br>*pounds (lbs)*	text (number, Min: 60, Max: 600), Required
	20	[sex]	What is your biological sex at birth?	radio, Required<br>1 Male<br>2 Female
	21	[gender]	What gender do you identify with?	radio, Required<br>1 Female<br>2 Male<br>3 Transgender Female<br>4 Transgender Male<br>5 Gender Variant/Non-Conforming<br>6 Not listed
	22	[race]	What is your race? Check all that apply)	checkbox, Required, Identi er<br>1 race___1 American Indian<br>2 race___2 Alaska Native<br>3 race___3 Asian<br>4 race___4 Black or African American<br>5 race___5 Native Hawaiian or Other Paci c Islander<br>6 race___6 White
	23	[ethnicity]	What is your ethnicity?	radio, Required<br>1 Hispanic or Latino<br>2 Non-Hispanic or Latino<br>3 Prefer not to answer
	24	[income]	Please indicate your current pre-tax household income.	radio, Required<br>1 $0 to 9,999<br>2 $10,000 to 19,999<br>3 $20,000 to 49,999<br>4 $50,000 to 99,999

				5 | $100,000 or more
	25	[education]	What is the highest level of education you have obtained?	radio, Required<br>1 | Some high school<br>2 | High school diploma or GED<br>3 | Trade School<br>4 | Associate's Degree<br>5 | Bachelor's Degree<br>6 | Master's Degree<br>7 | Ph.D. or Professional Degree
	26	[demo_height_cm]	Height converted to cm	calc<br>Calculation: [demo_height]*2.54<br>Field Annotation: @HIDDEN-SURVEY
	27	[demo_weight_kg]	weight converted to kg	calc<br>Calculation: [weight_selfreport]/2.2<br>Field Annotation: @HIDDEN-SURVEY
	28	[bmi_selfreport]	BMI	calc<br>Calculation:<br>round(([demo_weight_kg]*10000)/(([demo_heigh 1)<br>Field Annotation: @HIDDEN-SURVEY
	29	[demographics_complete]	Section Header: *Form Status*<br>Complete?	dropdown<br>0 | Incomplete<br>1 | Unveri ed<br>2 | Complete

Instrument: Pre educational survey (pre_educational_survey) Enabled as survey

	30	[pre_order]	Section Header: *In the last week, how many days (0-7) did you:*<br>Eat a meal or food from a restaurant/market/convenient store (either dine in or take out)?	dropdown, Required<br>0 | 0<br>1 | 1<br>2 | 2<br>3 | 3<br>4 | 4<br>5 | 5<br>6 | 6<br>7 | 7

	31	[pre_pkg]	Prepare a meal or food with prepackaged foods only (i.e. ready - to-eat meals/frozen dinners/prepackaged meal solutions)?	dropdown, Required<br>0 0<br>1 1<br>2 2<br>3 3<br>4 4<br>5 5<br>6 6<br>7 7
	32	[pre_frozen]	Prepare a meal or food with prepackaged ingredients only (i.e. prepackaged ingredients like frozen peas, canned beans, jar of pasta sauce etc.)?	dropdown, Required<br>0 0<br>1 1<br>2 2<br>3 3<br>4 4<br>5 5<br>6 6<br>7 7
	33	[pre_scratch]	Prepare a meal or food from scratch at home?	dropdown, Required<br>0 0<br>1 1<br>2 2<br>3 3<br>4 4<br>5 5<br>6 6<br>7 7
	34	[pre_salt]	Prepare a meal or food using salt?	dropdown, Required<br>0 0<br>1 1<br>2 2

				3 3<br>4 4<br>5 5<br>6 6<br>7 7
	35	[pre_sugar]	Prepare a meal or food using added sugar (i.e. sugar, honey, syrup)?	dropdown, Required<br>0 0<br>1 1<br>2 2<br>3 3<br>4 4<br>5 5<br>6 6<br>7 7
	36	[pre_fat]	Prepare a meal or food using a fat that is high in saturated fat (i.e. butter, lard, cream)?	dropdown, Required<br>0 0<br>1 1<br>2 2<br>3 3<br>4 4<br>5 5<br>6 6<br>7 7
	37	[pre_spice]	Prepare a meal or food using herbs and spices for seasoning?	dropdown, Required<br>0 0<br>1 1<br>2 2<br>3 3<br>4 4<br>5 5

				6 6<br>7 7
	38	[pre_educational_survey_complete]	Section Header: *Form Status*<br>Complete?	dropdown<br>0 Incomplete<br>1 Unveri ed<br>2 Complete

Instrument: Interest, knowledge, and con dence (interest_knowledge_and_con dence) ▯ Enabled as survey

	39	[pre_interest1]	Section Header: *The next few questions ask about your interest, knowledge and con dence in using herbs and spices when cooking.*<br>What is your level of interest in the avor pro les of herbs and spices?	radio, Required<br>1 Extremely disinterested<br>2 Somewhat disinterested<br>3 Neither disinterested nor interested<br>4 Somewhat interested<br>5 Extremely interested
	40	[pre_interest2]	What is your level of interest in incorporating herbs and spices into your cooking?	radio, Required<br>1 Extremely disinterested<br>2 Somewhat disinterested<br>3 Neither disinterested nor interested<br>4 Somewhat interested<br>5 Extremely interested
	41	[pre_interest3]	What is your level of interest in using herbs and spices to increase your consumption of healthier foods?	radio, Required<br>1 Extremely disinterested<br>2 Somewhat disinterested<br>3 Neither disinterested nor interested<br>4 Somewhat interested<br>5 Extremely interested
	42	[pre_interest4]	What is your level of interest in using herbs and spices to decrease the use of salt in your cooking?	radio, Required<br>1 Extremely disinterested<br>2 Somewhat disinterested<br>3 Neither disinterested nor interested

				4 Somewhat interested<br>5 Extremely interested
	43	[pre_interest5]	What is your level of interest in using herbs and spices to decrease the use of added sugar in your cooking?	radio, Required<br>1 Extremely disinterested<br>2 Somewhat disinterested<br>3 Neither disinterested nor interested<br>4 Somewhat interested<br>5 Extremely interested
	44	[pre_interest6]	What is your level of interest in using herbs and spices to decrease the use of fats high in saturated fat (i.e. butter, lard, cream) in your cooking?	radio, Required<br>1 Extremely disinterested<br>2 Somewhat disinterested<br>3 Neither disinterested nor interested

				4 Somewhat interested<br>5 Extremely interested
	45	[pre_know1]	Section Header:<br>Please indicate your level of knowledge in using herbs and spices when preparing foods or a meal.	radio, Required<br>1 Very unknowledgeable<br>2 Somewhat unknowledgeable<br>3 Neutral<br>4 Somewhat knowledgeable<br>5 Very knowledgeable
	46	[pre_know2]	Please indicate your level of knowledge in making food healthier by avoring foods/ meals with herbs and spices as a substitute for salt, added sugar, and fats high in saturated fat (i.e. butter, lard, cream).	radio, Required<br>1 Very unknowledgeable<br>2 Somewhat unknowledgeable<br>3 Neutral<br>4 Somewhat knowledgeable<br>5 Very knowledgeable
	47	[pre_conf1]	Section Header:<br>Please indicate your level of con dence in using herbs and spices to decrease the amount of salt you use when preparing a meal or food.	radio, Required<br>1 Not con dent<br>2 Somewhat not con dent

	#	Variable	Field Label	Field Attributes
				3 Neutral<br>4 Somewhat con dent<br>5 Very con dent
	48	[pre_conf2]	Please indicate your level of con dence in using herbs and spices to decrease the amount of added sugar you use when preparing a meal or food.	radio, Required<br>1 Not con dent<br>2 Somewhat not con dent<br>3 Neutral<br>4 Somewhat con dent<br>5 Very con dent
	49	[pre_conf3]	Please indicate your level of con dence in using herbs and spices to decrease the amount of fats high in saturated fat (i.e. butter, lard, cream) you use when preparing a meal or food.	radio, Required<br>1 Not con dent<br>2 Somewhat not con dent<br>3 Neutral<br>4 Somewhat con dent<br>5 Very con dent
	50	[pre_conf4]	Please indicate your level of con dence in using herbs and spices to increase the palatability and consumption of healthier foods.	radio, Required<br>1 Not con dent<br>2 Somewhat not con dent<br>3 Neutral<br>4 Somewhat con dent<br>5 Very con dent
	51	[focus_check]	Section Header:<br>Please select 'strongly agree' to show you are paying attention to this question:	dropdown, Required<br>1 Agree<br>2 Disagree<br>3 Strongly disagree<br>4 Strongly agree<br>5 Neutral<br>Stop actions on 1, 2, 3, 5
	52	[subsalt]		radio, Required

			How likely are you to use herbs and spices as a substitute for salt when preparing your meals or food?	1 Very unlikely<br>2 Somewhat unlikely<br>3 Neutral<br>4 Somewhat likely<br>5 Very likely
	53	[subsugar]	How likely are you to use herbs and spices as a substitute for added sugar (i.e. sugar, honey, syrup) when preparing your meals or food?	radio, Required<br>1 Very unlikely<br>2 Somewhat unlikely<br>3 Neutral<br>4 Somewhat likely<br>5 Very likely
	54	[subfat]	How likely are you to use herbs and spices as a substitute for saturated fat (i.e. butter, lard, cream) when preparing your meals or food?	radio, Required<br>1 Very unlikely<br>2 Somewhat unlikely<br>3 Neutral<br>4 Somewhat likely<br>5 Very likely
	55	[subimprove]	How likely are you to use herbs and spices to improve the taste, and your consumption, of vegetables?	radio, Required<br>1 Very unlikely<br>2 Somewhat unlikely<br>3 Neutral<br>4 Somewhat likely<br>5 Very likely
	56	[mostused]	Please list the top 5 herbs and/ or spices you use most often when preparing your meals or food.	notes, Required<br>Custom alignment: RH
	57	[spicereason]	Please explain why do you use herbs and spices when preparing your meals or food.	notes, Required<br>Custom alignment: RH
	58	[proctor1]	I am in favor of using herbs and spices in my food	radio, Required<br>1 Strongly disagree<br>2 Disagree

			3 Neither agree nor disagree<br>4 Agree<br>5 Strongly agree
59	[proctor2]	I think using herbs and spices ts easily into my cooking	radio, Required<br>1 Strongly disagree<br>2 Disagree<br>3 Neither agree nor disagree<br>4 Agree<br>5 Strongly agree
60	[proctor3]	How often have you used herbs and spices in your cooking in the last month?	radio, Required<br>1 Never<br>2 Rarely<br>3 Sometimes<br>4 Often<br>5 Always
61	[interest_knowledge_and_confidence_complete]	Section Header: *Form Status*<br>Complete?	dropdown<br>0 Incomplete<br>1 Unveri ed<br>2 Complete
**Instrument: Educational Materials (educational_materials) ? Enabled as survey**			
62	[randomization]	Group Sorting	calc<br>Calculation: [record_id] - (rounddown([record_id]/2) * 2)<br>Field Annotation: @HIDDEN
63	[shortvid1]<br>Show the eld ONLY if:<br>[randomization] = '0'	Section Header: *Now, you will be presented with 5 videos pertaining to the use of herbs and spices while cooking.*<br>Please click to watch.	descriptive<br>(Media URL: https://youtu.be/8OSBzy9JVic, Display format: Inline)
64	[cc_vid1]<br>Show the eld ONLY if:<br>[randomization] = '0'	Section Header:<br>What color did you see at the end of video 1?	radio, Required<br>1 Yellow<br>2 Pink<br>3 Blue

65	[shortvid2]<br>Show the eld ONLY if:<br>[randomization] = '0'	Please click to watch.	descriptive<br>(Media URL: https://youtu.be/Iw73zy-NJ7M, Display format: Inline)
66	[cc_vid2]<br>Show the eld ONLY if:<br>[randomization] = '0'	Section Header:<br>What color did you see at the end of video 1?	radio, Required<br>1 Green<br>2 Red<br>3 Blue
67	[shortvid3]<br>Show the eld ONLY if:<br>[randomization] = '0'	Please click to watch.	descriptive<br>(Media URL: https://youtu.be/iCtGWY3uo_Q, Display format: Inline)
68	[cc_vid3]<br>Show the eld ONLY if:<br>[randomization] = '0'	Section Header:<br>What color did you see at the end of video 3?	radio, Required<br>1 Blue<br>2 Pink<br>3 Yellow
69	[shortvid4]<br>Show the eld ONLY if:<br>[randomization] = '0'	Please click to watch.	descriptive<br>(Media URL: https://youtu.be/jOXcCbEP79A, Display format: Inline)
70	[cc_vid4]<br>Show the eld ONLY if:<br>[randomization] = '0'	Section Header:<br>What color did you see at the end of video 4?	radio, Required<br>1 Red<br>2 Pink<br>3 Green
71	[shortvid5]<br>Show the eld ONLY if:<br>[randomization] = '0'	Please click to watch.	descriptive<br>(Media URL: https://youtu.be/T4jKnHhhUw4, Display format: Inline)
72	[cc_vid5]<br>Show the eld ONLY if:<br>[randomization] = '0'	What color did you see at the end of video 5?	radio, Required<br>1 Green<br>2 Yellow<br>3 Blue
73	[longvid1]<br>Show the eld ONLY if:<br>[randomization] = '1'	Section Header: *Now, you will be presented with 5 videos pertaining to the use of herbs and spices while cooking.*<br>Please click to watch.	descriptive<br>(Media URL: https://youtu.be/hC8uyt6dVhY, Display format: Inline)
74	[cc_vid6]<br>Show the eld ONLY if:<br>[randomization] = '1'	What color did you see at the end of video 1?	radio, Required<br>1 Yellow<br>2 Pink

			3 Red
75	[longvid2]<br><br>Show the eld ONLY if:<br>[randomization] = '1'	Section Header:<br>Please click to watch.	descriptive<br>(Media URL: https://youtu.be/Ii0tRnyxdUs, Display format: Inline)
76	[cc_vid7]<br><br>Show the eld ONLY if:<br>[randomization] = '1'	What color did you see at the end of video 2?	radio, Required<br>1 Blue<br>2 Orange<br>3 Yellow
77	[longvid3]<br><br>Show the eld ONLY if:<br>[randomization] = '1'	Section Header:<br>Please click to watch.	descriptive<br>(Media URL: https://youtu.be/hQa7sQA6Its, Display format: Inline)
78	[cc_vid8]<br><br>Show the eld ONLY if:<br>[randomization] = '1'	What color did you see at the end of video 3?	radio, Required<br>1 Pink<br>2 Blue<br>3 Yellow
79	[longvid4]<br><br>Show the eld ONLY if:<br>[randomization] = '1'	Section Header:<br>Please click to watch.	descriptive<br>(Media URL: https://youtu.be/jouARlSfSXo, Display format: Inline)
80	[cc_vid9]<br><br>Show the eld ONLY if:<br>[randomization] = '1'	What color did you see at the end of video 4?	radio, Required<br>1 Pink<br>2 Green<br>3 Red
81	[longvid5]<br><br>Show the eld ONLY if:<br>[randomization] = '1'	Section Header:<br>Please click to watch.	descriptive<br>(Media URL: https://youtu.be/pB-VeFhA5v4, Display format: Inline)
82	[cc_vid10]<br><br>Show the eld ONLY if:<br>[randomization] = '1'	What color did you see at the end of video 5?	radio, Required<br>1 Orange<br>2 Yellow<br>3 Blue
83	[materials_view]	Section Header: *Please answer the following question and click "Submit" only once all materials have been viewed. You will NOT be able to return to this portion of the survey.*<br><br>Did you view all the educational materials presented in this survey?	yesno, Required<br>1 Yes<br>0 No
84	[educational_materials_complete]	Section Header: *Form Status*<br><br>Complete?	dropdown<br>0 Incomplete

				1 Unveri ed<br>2 Complete

Instrument: **Post Interest, knowledge, and con dence** (post_interest_knowledge_and_con dence) ? **Enabled as survey**

	85	[post_interest1]	Section Header: *The next few questions ask about your interest, knowledge and con dence in using herbs and spices when cooking.*<br><br>After reviewing the materials provided, what is your level of interest in the avor pro les of herbs and spices?	radio, Required<br>1 Extremely disinterested<br>2 Somewhat disinterested<br>3 Neither disinterested nor interested<br>4 Somewhat interested<br>5 Extremely interested<br>Custom alignment: RV
	86	[post_interest2]	After reviewing the materials provided, what is your level of interest in incorporating herbs and spices into your cooking?	radio, Required<br>1 Extremely disinterested<br>2 Somewhat disinterested<br>3 Neither disinterested nor interested<br>4 Somewhat interested<br>5 Extremely interested<br>Custom alignment: RV
	87	[post_interest3]	After reviewing the materials provided, what is your level of interest in using herbs and spices to increase your consumption of healthier foods?	radio, Required<br>1 Extremely disinterested<br>2 Somewhat disinterested<br>3 Neither disinterested nor interested<br>4 Somewhat interested<br>5 Extremely interested<br>Custom alignment: RV
	88	[post_interest4]	After reviewing the materials provided, what is your level of interest in using herbs and spices to decrease the use of salt in your cooking?	radio, Required<br>1 Extremely disinterested<br>2 Somewhat disinterested<br>3 Neither disinterested nor interested<br>4 Somewhat interested<br>5 Extremely interested<br>Custom alignment: RV

	89	[post_interest5]	After reviewing the materials provided, what is your level of interest in using herbs and spices to decrease the use of added sugar in your cooking?	radio, Required<br>1 Extremely disinterested<br>2 Somewhat disinterested<br>3 Neither disinterested nor interested<br>4 Somewhat interested<br>5 Extremely interested<br>Custom alignment: RV
	90	[post_interest6]	After reviewing the materials provided, what is your level of interest in using herbs and spices to decrease the use of fats high in saturated fat (i.e. butter, lard, cream) in your cooking?	radio, Required<br>1 Extremely disinterested<br>2 Somewhat disinterested<br>3 Neither disinterested nor interested<br>4 Somewhat interested<br>5 Extremely interested<br>Custom alignment: RV
	91	[post_know1]	Section Header:<br>After reviewing the materials provided, indicate your level of knowledge in using herbs and spices when preparing a meal or food.	radio, Required<br>1 Very unknowledgeable<br>2 Somewhat unknowledgeable<br>3 Neutral<br>4 Somewhat knowledgeable<br>5 Very knowledgeable<br>Custom alignment: RV
	92	[post_know2]	After reviewing the materials provided, indicate your level of knowledge in making food healthier by avoring meals or food with herbs and spices as a substitute for salt, added sugar, and fats high in saturated fat (i.e. butter, lard, cream).	radio, Required<br>1 Very unknowledgeable<br>2 Somewhat unknowledgeable<br>3 Neutral<br>4 Somewhat knowledgeable<br>5 Very knowledgeable<br>Custom alignment: RV
	93	[post_conf1]	Section Header:<br>After reviewing the materials provided, indicate your level of con dence in using herbs and spices to decrease the amount of salt you use when preparing a meal or food.	radio, Required<br>1 Not con dent<br>2 Somewhat not con dent<br>3 Neutral<br>4 Somewhat con dent<br>5 Very con dent

				Custom alignment: RV
	94	[post_conf2]	After reviewing the materials provided, indicate your level of con dence in using herbs and spices to decrease the amount of added sugar (i.e. sugar, honey, syrup) you use when preparing a meal or food.	radio, Required<br>1 Not con dent<br>2 Somewhat not con dent<br>3 Neutral<br>4 Somewhat con dent<br>5 Very con dent<br>Custom alignment: RV
	95	[post_conf3]	After reviewing the materials provided, indicate your level of con dence in using herbs and spices to decrease the amount of fats high in saturated fat (i.e. butter, lard, cream) you use when preparing a meal or food.	radio, Required<br>1 Not con dent<br>2 Somewhat not con dent<br>3 Neutral<br>4 Somewhat con dent<br>5 Very con dent<br>Custom alignment: RV
	96	[post_conf4]	After reviewing the materials provided, indicate your level of con dence in using herbs and spices to increase the palatability and consumption of healthier foods.	radio, Required<br>1 Not con dent<br>2 Somewhat not con dent<br>3 Neutral<br>4 Somewhat con dent<br>5 Very con dent<br>Custom alignment: RV

	97	[focus_check_2]	Please select 'neutral' to show you are paying attention to this question:	dropdown, Required<br>1 Agree<br>2 Neutral<br>3 Strongly disagree<br>4 Strongly agree<br>5 Disagree<br>Stop actions on 1, 3, 4, 5
	98	[postsubsalt]	Section Header:<br>After reviewing the materials provided, how likely are you to use herbs and spices as a substitute for salt when preparing your meals or food?	radio, Required<br>1 Very unlikely<br>2 Somewhat unlikely<br>3 Neutral<br>4 Somewhat likely<br>5 Very likely<br>Custom alignment: RV
	99	[postsubsugar]	After reviewing the materials provided, how likely are you to use herbs and spices as a substitute for added sugar (i.e. sugar, honey, syrup) when preparing your meals or food?	radio, Required<br>1 Very unlikely<br>2 Somewhat unlikely<br>3 Neutral<br>4 Somewhat likely<br>5 Very likely<br>Custom alignment: RV

	100	[postsubfat]	After reviewing the materials provided, how likely are you to use herbs and spices as a substitute for saturated fat (i.e. butter, lard, cream) when preparing your meals or food?	radio, Required<br>1 Very unlikely<br>2 Somewhat unlikely<br>3 Neutral<br>4 Somewhat likely<br>5 Very likely<br>Custom alignment: RV

	101	[postsubimprove]	After reviewing the materials provided, how likely are you to use herbs and spices to improve the taste, and your consumption, of vegetables?	radio, Required<br>1 Very unlikely<br>2 Somewhat unlikely<br>3 Neutral<br>4 Somewhat likely<br>5 Very likely<br>Custom alignment: RV
	102	[postbarriers]	After reviewing the materials provided, do you believe there will be any barriers that may limit you from using herbs and spices when cooking in the future?	yesno<br>1 Yes<br>0 No
	103	[postqual_barriers]<br>Show the eld ONLY if:<br>[postbarriers] = '1'	In your own words, please explain what are the barriers that may limit you from using herbs and spices in your cooking in the future:	notes
	104	[post_interest_knowledge_and_confidence_complete]	Section Header: *Form Status*<br>Complete?	dropdown<br>0 Incomplete<br>1 Unveri ed<br>2 Complete

Instrument: **Matrix Reasons**
(matrix_reasons) **Enabled as survey**

	105	[postreason1]	Section Header: *After reviewing the materials provided, someone may still not want to or may be unable to use herbs and spices in their cooking. For the reasons listed below, please indicate whether you feel each may still be a major reason, minor reason, or not a reason for why you may not want to or may be unable to use herbs and spices in your cooking.*<br>I feel that herbs and spices have distinctive avors that are too strong for me	radio (Matrix)<br>1 Not a reason<br>2 Minor reason<br>3 Major reason
	106	[postreason2]	I feel that herbs and spices have distinctive avors that are too strong for my family members	radio (Matrix)<br>1 Not a reason<br>2 Minor reason<br>3 Major reason
	107	[postreason3]	I think herbs and spices are expensive to purchase	radio (Matrix)<br>1 Not a reason<br>2 Minor reason<br>3 Major reason

	108	[postreason4]	I do not have the time to incorporate herbs and spices into my cooking	radio (Matrix)<br>1 Not a reason<br>2 Minor reason<br>3 Major reason
	109	[postreason5]	I do not have the knowledge on how to use herbs and spices in my cooking	radio (Matrix)<br>1 Not a reason<br>2 Minor reason<br>3 Major reason
	110	[postreason6]	I do not have access to herbs and spices where I live	radio (Matrix)<br>1 Not a reason<br>2 Minor reason<br>3 Major reason
	111	[postreason7]	I feel that the people I cook for will not like herbs and spices being added to foods and meals	radio (Matrix)<br>1 Not a reason<br>2 Minor reason<br>3 Major reason
	112	[postreason8]	I am unable to make changes in the way I cook now	radio (Matrix)<br>1 Not a reason<br>2 Minor reason<br>3 Major reason
	113	[postproctor1]	After reviewing the materials provided, I am in favor of using herbs and spices in my food	radio<br>1 Strongly disagree<br>2 Disagree<br>3 Neither agree or disagree<br>4 Agree<br>5 Strongly agree
	114	[postproctor2]	After reviewing the materials provided, I think using herbs and spices ts easily into my cooking	radio<br>1 Strongly disagree<br>2 Disagree

				3 Neither agree or disagree<br>4 Agree<br>5 Strongly agree
	115	[postproctor3]	After reviewing the materials provided, how often will you try using herbs and spices in your cooking in the next month?	radio<br>1 Never<br>2 Rarely<br>3 Sometimes<br>4 Often<br>5 Always
	116	[matrix_reasons_complete]	Section Header: *Form Status*<br>Complete?	dropdown<br>0 Incomplete<br>1 Unveri ed<br>2 Complete

Instrument: **Evaluation Questions** (evaluation_questions) ? Enabled as survey

	117	[eval1]	Section Header: *Please respond with how much you agree or disagree with the following statements: The nutrition education videos...*<br>Conveyed educational information about the use of herbs and spices when cooking	radio<br>1 Strongly disagree<br>2 Disagree<br>3 Neither agree or disagree<br>4 Agree<br>5 Strongly agree
	118	[eval2]	Provided video content in a concise manner regarding the use of herbs and spices when cooking	radio<br>1 Strongly disagree<br>2 Disagree<br>3 Neither agree or disagree<br>4 Agree<br>5 Strongly agree
	119	[eval3]	Provided me with credible information on how to use herbs and spices when cooking	radio<br>1 Strongly disagree<br>2 Disagree

				3 Neither agree or disagree<br>4 Agree<br>5 Strongly agree
	120	[eval4]	Were easy to follow and understand	radio<br>1 Strongly disagree<br>2 Disagree<br>3 Neither agree or disagree<br>4 Agree<br>5 Strongly agree
	121	[eval5]	Provided me with relevant information on how to increase the use of herbs and spices in my cooking	radio<br>1 Strongly disagree<br>2 Disagree<br>3 Neither agree or disagree<br>4 Agree<br>5 Strongly agree
	122	[eval6]	Encouraged me to use herbs and spices in my cooking moving forward	radio<br>1 Strongly disagree<br>2 Disagree<br>3 Neither agree or disagree<br>4 Agree<br>5 Strongly agree
	123	[eval7]	Were an e ective method to help increase the use of herbs and spices in my cooking	radio<br>1 Strongly disagree<br>2 Disagree<br>3 Neither agree or disagree<br>4 Agree<br>5 Strongly agree
	124	[eval8]	Were interesting and engaging	radio<br>1 Strongly disagree

				2 Disagree<br>3 Neither agree or disagree<br>4 Agree<br>5 Strongly agree
	125	[evaluation_questions_complete]	Section Header: *Form Status*<br>Complete?	dropdown<br>0 Incomplete<br>1 Unveri ed<br>2 Complete

Instrument: **Video Feedback** (video_feedback) ? **Enabled as survey**

	126	[improve1]	Section Header: *The following questions inquire about improvements you would make to these nutrition education videos:*<br>What would you add to the existing nutrition education video content?	notes
	127	[info1]	Is there more information about herbs and spices you would like to see in future videos?	yesno<br>1 Yes<br>0 No
	128	[info2]<br>Show the eld ONLY if: [info1] = "1"	If yes, please explain:	notes
	129	[info3]	Would you like to learn more about using spices in recipes from other cuisines?	yesno<br>1 Yes<br>0 No
	130	[info4]<br>Show the eld ONLY if: [info3] = '1'	What recipes from other regions would you like to learn about?	checkbox<br>1 info4___1 Mexican cuisine<br>2 info4___2 Asian cuisine (Thai, Chinese, Korean, Japanese)<br>3 info4___3 Indian cuisine<br>4 info4___4 Mediterranean cuisine (Greek, Arabic)<br>5 info4___5 Italian cuisine<br>6 info4___6 Caribbean cuisine (Jamaican)<br>7 info4___7 Other

131	[info5]<br><br>Show the eld ONLY if:<br>[info4(7)] = '1'	If other, please explain	notes
132	[spice_learn1]	Why do you want to learn more about herbs and spices?	checkbox<br><table><tr><td>1</td><td>spice_learn1___1</td><td>Cooking with spices is familiar to me</td></tr><tr><td>2</td><td>spice_learn1___2</td><td>Food tastes better with spices</td></tr><tr><td>3</td><td>spice_learn1___3</td><td>Spices are a part of my culture</td></tr><tr><td>4</td><td>spice_learn1___4</td><td>Other</td></tr></table>
133	[spice_learn2]<br><br>Show the eld ONLY if:<br>[spice_learn1(4)] = '1'	If other, please explain	text
134	[info6]	What other spices would you like to see included in our recipes?	notes
135	[info7]	Do you have any other feedback regarding the questions/content of these educational videos?	notes
136	[increase_veggies]	Section Header:<br><br>After reviewing the provided materials, do you believe using herbs and spices in your cooking will help you increase your consumption of vegetables?	notes, Required<br>Custom alignment: RH
137	[flavor_veggies]	After reviewing the provided materials, how likely are you to use herbs and spices to improve the taste, and your consumption, of vegetables?	notes, Required<br>Custom alignment: RH
138	[new_spices]	While reviewing the provided materials, did you learn about any new herbs or spices?	dropdown, Required<br><table><tr><td>1</td><td>Yes</td></tr><tr><td>2</td><td>No</td></tr></table>Custom alignment: RH
139	[new_spices2]<br><br>Show the eld ONLY if:<br>[new_spices] = '1'	Please list the new herbs or spices you learned about.	notes, Required<br>Custom alignment: RH
140	[top_5_spices]	After having reviewed the provided materials, please list the top 5 herbs and spices you anticipate to use when preparing a meal or food.	notes, Required<br>Custom alignment: RH
141	[video_feedback_complete]	Section Header: *Form Status*<br><br>Complete?	dropdown<br><table><tr><td>0</td><td>Incomplete</td></tr><tr><td>1</td><td>Unveri ed</td></tr></table>

				2 Complete

Instrument: **Engagement Questionnaire** (engagement_questionnaire) ? Enabled as survey

	142	[eq_info]	Please rate the extent you agree with each of the following statements.	descriptive
	143	[eq_2]	I lost interest in the videos	radio, Required<br>7 Strongly disagree<br>6 Disagree<br>5 Somewhat disagree<br>4 Neutral<br>3 Somewhat agree<br>2 Agree<br>1 Strongly agree
	144	[eq_3]	I was distracted	radio, Required<br>7 Strongly disagree<br>6 Disagree<br>5 Somewhat disagree<br>4 Neutral<br>3 Somewhat agree<br>2 Agree<br>1 Strongly agree
	145	[eq_1]	I felt myself zoning out during videos	radio, Required<br>7 Strongly disagree<br>6 Disagree<br>5 Somewhat disagree<br>4 Neutral<br>3 Somewhat agree<br>2 Agree<br>1 Strongly agree
	146	[eq_7]	I found the videos meaningful	radio

				1 Strongly disagree<br>2 Disagree<br>3 Somewhat disagree<br>4 Neutral<br>5 Somewhat agree<br>6 Agree<br>7 Strongly agree
	147	[eq_4]	I felt dedicated to nish the videos	radio, Required<br>1 Strongly disagree<br>2 Disagree<br>3 Somewhat disagree<br>4 Neutral<br>5 Somewhat agree<br>6 Agree<br>7 Strongly agree
	148	[eq_6]	I wanted to devote my full attention to the videos	radio, Required<br>1 Strongly disagree<br>2 Disagree<br>3 Somewhat disagree<br>4 Neutral<br>5 Somewhat agree<br>6 Agree<br>7 Strongly agree
	149	[eq_9]	I found the videos captivating	radio, Required<br>1 Strongly disagree<br>2 Disagree<br>3 Somewhat disagree<br>4 Neutral<br>5 Somewhat agree

				6 Agree<br>7 Strongly agree
	150	[eq_8]	I enjoyed the videos	radio, Required<br>1 Strongly disagree<br>2 Disagree<br>3 Somewhat disagree<br>4 Neutral<br>5 Somewhat agree<br>6 Agree<br>7 Strongly agree
	151	[enjoy_vid]	Section Header: *On a scale of 1-100,*<br>How much did you like the nutrition education videos?	slider (number, Min: 0, Max: 100), Required<br>Slider labels: Did not like them, , Loved them<br>Custom alignment: RH
	152	[engagement_questionnaire_complete]	Section Header: *Form Status*<br>Complete?	dropdown<br>0 Incomplete<br>1 Unveri ed<br>2 Complete
