# Supplementary figures and images for: Nutrition Education “Shorts”: The Effect of Short-Form Media on Conveying Information About Improving Diet Quality
Source: Nutrients. 2025 May 8;17(10):1612. doi: 10.3390/nu17101612 (PMC12114486; doi:10.3390/nu17101612)

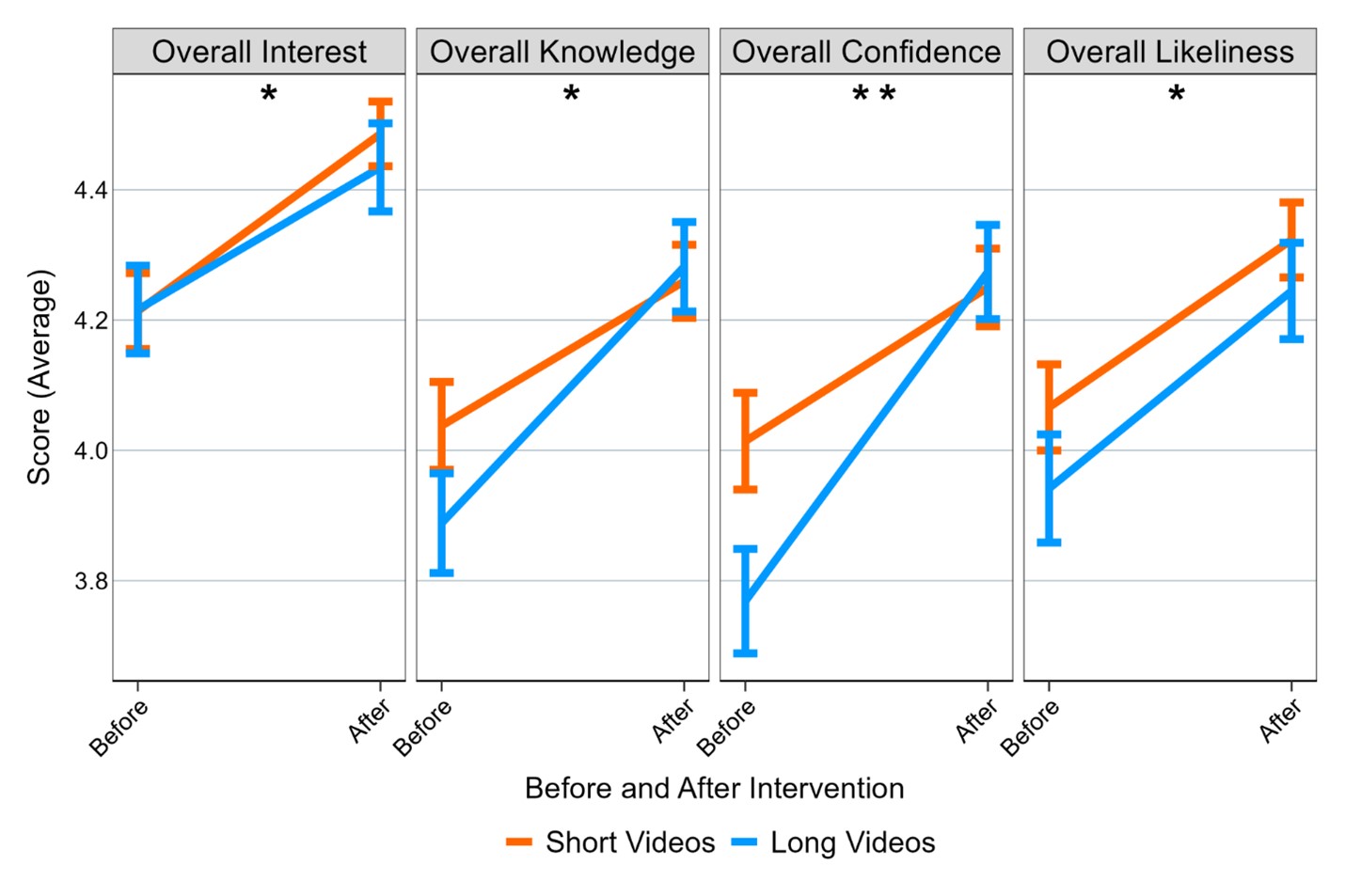

Supplement: Supplementary file 1 [file nutrients-17-01612-s001.zip › Supplementary Figure S1.jpg]

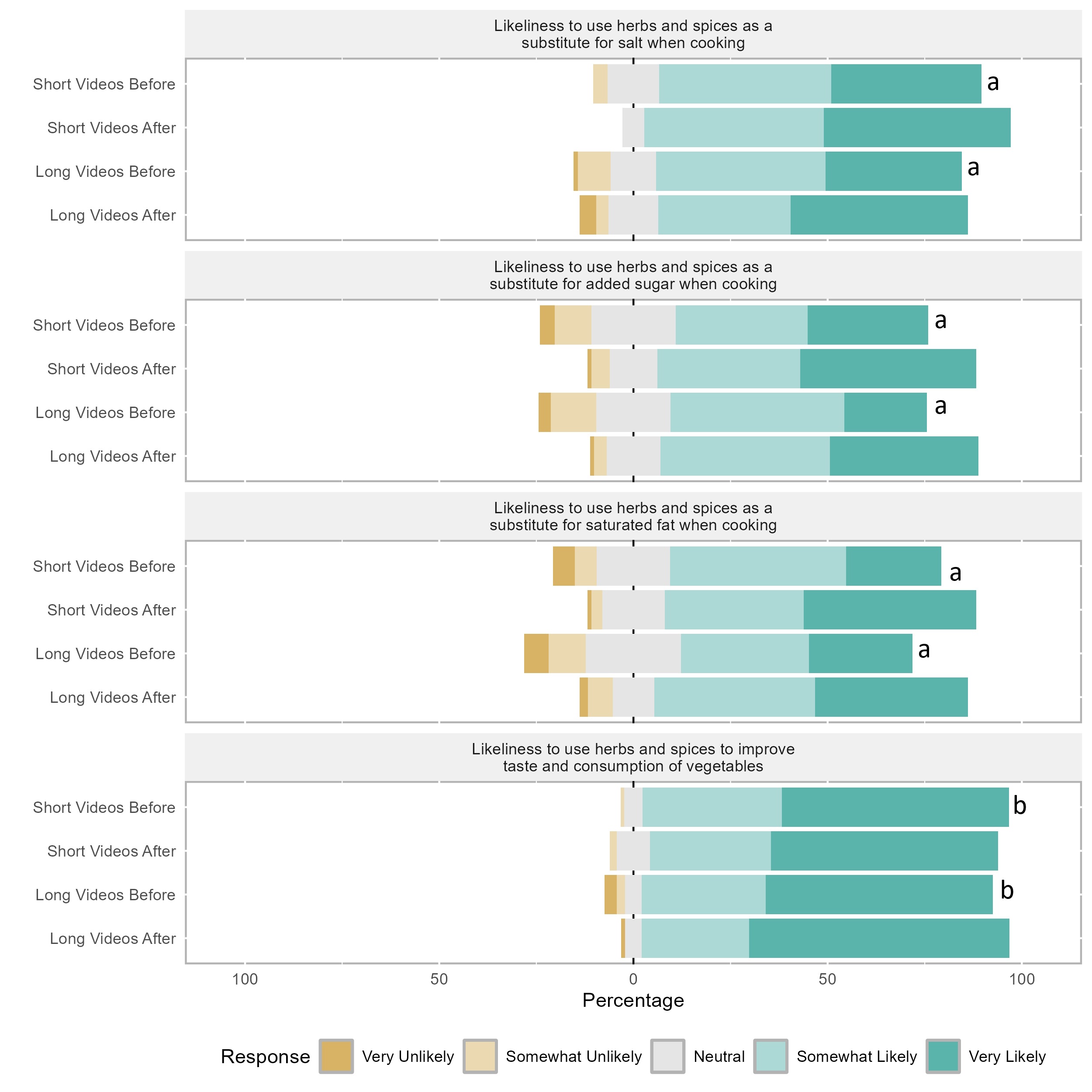

Supplement: Supplementary file 1 [file nutrients-17-01612-s001.zip › Supplementary_Figure_S2.jpg]
